# Editorial: Novel methodologies in structural interventional cardiology: Case reports

**DOI:** 10.3389/fcvm.2023.1191819

**Published:** 2023-04-12

**Authors:** David O'Sullivan, Crochan J. O'Sullivan, Micha T. Maeder

**Affiliations:** ^1^ Cardiology Department, Mater Misericordiae University Hospital, Dublin, Ireland; ^2^ Cardiology Department, Triemli Hospital, Zurich, Switzerland; ^3^ Cardiology Department, Bon Secours Hospital Cork, Cork, Ireland; ^4^ Cardiology Department, Cantonal Hospital St.Gallen, St. Gallen, Switzerland

**Keywords:** structural interventional cardiology, TAVI – transcatheter aortic valve implantation, left atrial appendage (LAA) occlusion, 3D prinitng, TAVI complications, novel methodologies

**Editorial on the Research Topic** Novel methodologies in structural interventional cardiology: Case reports

Structural interventional cardiology has rapidly evolved since the innovation of coronary balloon angioplasty in 1977. Having initially been dominated by coronary intervention the field has grown to provide minimally invasive alternatives to traditional surgical procedures for the treatment of a range of cardiovascular diseases. The continuous advances in technology, new devices, procedural strategies, and imaging techniques that have emerged in the field has led to improved patient outcomes, reduced procedural complications, and expanded indications for interventional treatments. Many inoperable patients are now eligible for the correction of structural cardiac defects with benefits in terms of functional capacity and quality of life. At the forefront of innovation are procedures such as insertion of left atrial appendage occlusion (LAAO) devices, transcatheter aortic valve implantation (TAVI) as well as advancements in imaging techniques, such as multidetector computed tomography (MDCT) and three-dimensional (3D) reconstruction (1–4). In this editorial, we will discuss some of the latest advancements and novel methodologies in structural intervention by summarising the main findings and key aspects of a collection of articles published in Frontiers in Cardiovascular Medicine under this exciting research topic.

A key adjunct to structural interventional advances is the parallel evolution of cardiac imaging techniques and use of novel technologies such as 3D reconstruction. *Case Report: Severe Aortic Valve Regurgitation and Pseudoaneurysm in Aortic Valve-Sparing Operation: The usefulness of Multimodality imaging in a Complex Clinical Scenario*, by Leonardo Varotto et al. perfectly illustrates this point. The authors presented a case of a patient presenting with two potentially disastrous complications of aortic valve sparing surgery in that of severe aortic insufficiency and aortic arch pseudoaneurysm. They showed how the use of contrast-enhanced computed tomography and its translation into a 3D printed model was able to provide an accurate representation of the complex anatomy of the aortic root aneurysm and aortic root geometry. This enabled the team, with the aid of transoesophageal 3D ultrasound, to efficaciously treat the patient percutaneously with a combination of transcatheter occluder device plus microcoil embolization and transfemoral valve implantation. The use of multimodal imaging and formation of a 3D printed model in this complex case highlights how these techniques can aid in preoperational planning hopefully leading to the reduction of intra- and post-operative complications.

“*Catheter-Based Closure of a Post-infective Aortic Paravalvular Pseudoaneurysm Fistula with Severe Regurgitation After Two Valve Replacement Surgeries: A Case Report”* by Eustaquio Maria Onorato et al. continues the above theme of combining novel interventional and imaging techniques to tackle complex pathologies in patients at prohibitive surgical risk. Here, the authors report a case of a paravalvular pseudoaneurysm fistula (PPF) with severe paravalvular leak (PVL) after infective endocarditis of a bioprosthetic valve causing severe heart failure in a 72 year old female with multiple comorbidities. Again a 3D model is printed this time based on a MDCT reconstruction in order to better understand PPF anatomy and its relationship with the surrounding structures. This allowed for planning of “ex vivo” transcatheter implantation of the most appropriate occluding device to limit residual leak and prevent serious complications. Successful implantation of an Occlutech paravalvular leak device was achieved without any intra or postoperative complications perhaps indicating that catheter-based closure of PPF may be a feasible and safe alternative to surgical repair.

As shown in the previous cases advanced imaging modalities and 3D reconstruction can be a powerful adjunct to the structural interventional cardiologist. Despite this, complex anatomy can often be prohibitive to traditional interventional methodologies and thus a search for novel techniques is ever ongoing. Tao Chen et al. in their study entitled “*Occlusion of Bilobulated Left Atrial Appendage Using the Dual-Watchman Technique: A Long Term Follow-Up Study”* provides a good example of this, describing how the heterogeneous anatomy of the left atrial appendage has to date proved an obstacle for LAAO in a certain subset of cases. The authors conducted a retrospective analysis of 330 patients undergoing LAAO with watchman devices and found 7 cases where large bilobulated LAA's were successfully closed with dual watchman devices using one-stop implantation technology. Although a retrospective study with a small sample size, it provides an indicator that a dual device approach is feasible in a patient population with challenging anatomy where single device closure has been ruled out.

With the ongoing evolution of structural interventional techniques, such as those mentioned in the case reports thus far, the risk of complications inevitably rises. As such novel methodologies to mitigate these complications are as important to highlight in the literature. “*Case Report: Sapien 3 Transcatheter Heart Valve Embolization: Cause, Management, and Redo”* by Mi Chen et al. highlights a rare and challenging complication of TAVI in that of transcatheter heart valve (THV) embolization. The authors go on to describe the subsequent repositioning and successful navigation of the Sapien 3 THV through the aortic arch using a buddy balloon and performing a pullback manoeuvre into the descending aorta on the shoulder of the inflated deployment balloon. A second THV is then implanted with careful post-implantation evaluation.

Finally we finish with a case report by Xiangfei Haung et al. entitled “*Differential Diagnosis of Fulminant Myocarditis and Acute Coronary Syndromes in the Case of Failure of Coronary Angiography: A Case Report”*. The authors present the readers with a case of 34 year old male with fulminant myocarditis whose presentation is complicated by a coronary artery malformation resulting in failure of coronary angiography and thus initial delay in the true diagnosis. As a result of a comprehensive approach including careful history taking, adjunct imaging and supportive measures such as intra-aortic balloon pump insertion and vein-artery extracorporeal membrane oxygenation (ECMO) the patient was successfully treated and discharged home after 27 days in hospital.
